# The Relief of Pain from Diseases of the Dental Pulp and Peridental Membrane

**Published:** 1894-03

**Authors:** A. W. Harlan

**Affiliations:** Chicago, Ill.


ARTICLE V.

### THE RELIEF OF PAIN FROM DISEASES OF THE DENTAL PULP AND PERIDENTAL MEMBRANE.

BY A. W. HARLAN, M. D., D. D. S., CHICAGO, ILL.

The dental pulp is a frequent cause of pain after near or actual exposure of its surface to external agencies. When the pulp is exposed by accident the pain is easily relieved by protecting it from the air or moisture with chloral champhor, phenol camphor, oleate of cocaine, melted carbolic acid or other local anæsthetic. The surface should be dried if possible before making the application. A mixture of collodion and carbolic acid, ten or twenty per cent. will serve as a temporary dressing. Twenty parts of carbolic acid. five parts of the hydrochlorate of cocaine and seventy-five parts of liquid vaseline will arrest pain from exposure of the pulp. I have frequently used twenty parts of a four per cent. solution of cocaine, thirty parts of pure oil of sassafras and fifty parts of melted carbolic acid as a local covering. This is only slightly caustic or escharotic. The bottle should be shaken before using when the mixture is fresh. The pain from a hyperæmic pulp is quickly relieved by puncture, when possible, if not, torsion will sometimes relieve the pain. Remedies administered internally for retarding the circulation will seldom be effective in relieving the hyperæmic condition. Sometimes when torsion is practiced the addition of counter-irritation may relieve the pain. When the pulp of a tooth has been capped with any material before it is in a normal condition, there may be pain, continuous or intermittent. Should this continue in spite of torsion or counter-irritation, the filling must be removed. Even this will not always relieve the pain and the pulp may have to be destroyed before the pain will cease. Of course it is understood that a pulp should not be capped when irritated or inflamed, but many pulps are capped in this condition and the only salvation for them is to remove the capping.

The pain from a pulp where calcification of its substance is going on cannot be relieved permanently save by destruction of the organ. If the patient can endure the pain, in a course of time the pulp will be obliterated. Usually the patient will not endure such agony for very long, and unless relief is afforded, the tooth will be extracted by some other dentist.

The pain from congestion of the pulp and the formation of pus in its substance can only be relieved by getting direct access to it and pricking it to relieve the overfull vessels. After the tension has been relieved, the pain does not always cease. It has been a favorite method with me to wash the cavity with peroxide of hydrogen at once and quickly dry the cavity; apply pure chloroform on cotton, then melted carbolic acid. In five or ten minutes the patient will be comfortable. It is my theory to destroy the pulp if there is no probability of saving it. I might attempt to coax it back to health if it were an exposed front tooth in a young person's mouth. There are few cases where it is possible to retain the vitality of a pulp after suppuration of a small portion of its substance. The mere puncturing of a pulp with a sharp-pointed instrument to relieve congestion or hyperæmia is not always sufficient reason for the destruction of a pulp unless the vital powers are low or the patient is past fifty years of age; then the recuperative forces may not be sufficient to enable it to live under a capping. Drying the surface of a suddenly exposed pulp and painting it with collodion will arrest pain. When the pulp is dead and the pain results from pressure of pus beyond the apex, the manifest duty of the surgeon is to give it exit, through the root-canal or by drilling into the alveolus through the process. If a simple pericementitis has to be dealt with, calcium sulphide 1-10 gr. pill every ten minutes until six have been taken will ordinarily arrest pain. Prior to this an aperient may be administered. Citrate of magnesia, a Seidlitz powder, hunyadi janos, or some other internal remedy. Counter-irritation, tincture of capsicum, cantharides, ammonia, chloroform, absolute alcohol or a metal disc of the size and thickness of a copper cent dropped in boiling water before using it. When there is a pocket alongside the root, wash with pyrozone, then inject into the pocket two or three drops of vinum opii or a twenty per cent. solution of menthol in alcohol, or ten per cent. acid carbolic in liquid vaseline. Bathe the face in hot water, place towels or napkins on the face after dipping them in water at 140° F. Keep changing them and relief will come. When the pulp is exposed at the apex, destroy it.—*Ed. in Review.*

